# Case report: Diversity of imaging in cardiac angiosarcoma: two cases with disparate enhancement and metabolic manifestations

**DOI:** 10.3389/fonc.2025.1515950

**Published:** 2025-04-14

**Authors:** Sinan Chen, Huan Cen, Jie Zhao, Pengcheng Ran, Weihui Lu, Pengtao Sun

**Affiliations:** ^1^ Department of Ultrasonography, The Second Affiliated Hospital of Guangzhou University of Chinese Medicine, Guangzhou, China; ^2^ Department of Pathology, The Second Affiliated Hospital of Guangzhou University of Chinese Medicine, Guangzhou, China; ^3^ Department of Nuclear Medicine, The Second Affiliated Hospital of Guangzhou University of Chinese Medicine, Guangzhou, China; ^4^ Department of Cardiovascular Medicine, The Second Affiliated Hospital of Guangzhou University of Chinese Medicine, Guangzhou, China

**Keywords:** cardiac angiosarcoma, positron emission tomography, echocardiography, contrast-enhanced ultrasound, cardiac oncology

## Abstract

Cardiac angiosarcoma, a rare and aggressive malignancy arising from endothelial cells, is difficult to diagnose owing to its nonspecific clinical symptoms and variable imaging features. Two cases of cardiac angiosarcoma (CA) are presented, each with different enhancement and metabolic patterns on imaging. Case 1: A 59-year-old man presented with chest tightness and lower extremity edema. Ultrasound and computed tomography (CT) imaging revealed a hypoechoic/hypodense, non-enhancing mass with pericardial thickening in the right atrium. Positron emission tomography (PET) showed minimal uptake and, given that the patient had elevated D-dimer and fibrinogen levels, a thrombus was initially suspected. However, surgical intervention ultimately led to a diagnosis of CA. Case 2: A 27-year-old man presented with dyspnea and cough. Both ultrasound and CT imaging revealed a mass in the right atrium, with mid-to-low echogenic/hypodense features, heterogeneous enhancement, and pericardial effusion, along with pericardial thickening. A PET scan showed a significant increase in radiotracer uptake within the mass, strongly suggestive of CA. Surgical intervention subsequently confirmed the diagnosis of CA. These two cases demonstrate the presence of distinct enhancement and metabolic patterns on imaging in primary CA and indicate the importance of considering a wide range of enhancement features and metabolic activities in the differential diagnosis of patients presenting with non-specific cardiac symptoms.

## 1 Introduction

Cardiac angiosarcoma (CA), a rare and highly aggressive malignancy originating from endothelial cells, presents significant diagnostic challenges owing to its non-specific clinical manifestations and variable imaging features. Given its biological behavior and poor prognosis, early and accurate CA diagnosis is critical for optimizing therapeutic strategies and improving patient outcomes. Multimodal imaging tools, such as transthoracic echocardiography (TTE), contrast-enhanced ultrasound (CEUS), computed tomography angiography (CTA), and positron emission tomography (PET), are indispensable for the detection, characterization, and staging of CA. While CA typically exhibits hyperenhancement and hypermetabolism on imaging, this report presents two cases of CA with contrasting enhancement and metabolic patterns, highlighting the diversity of enhancement and metabolic pattern combinations in this malignancy. By presenting these cases, we aim to underscore the importance of considering a wide range of imaging and metabolic findings in the diagnosis and management of CA.

## 2 Case report

### 2.1 Case 1

**Figure 1:**
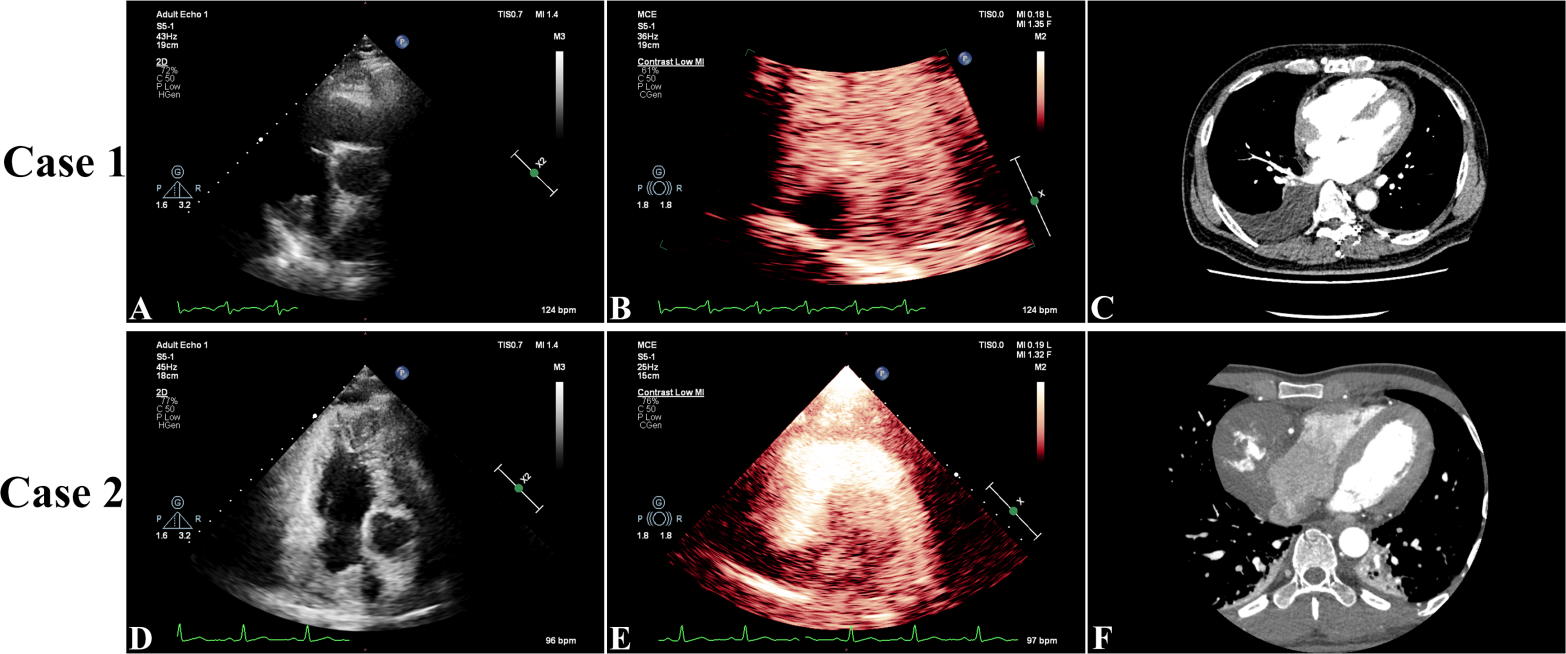
In Case 1, preoperative three-dimensional transthoracic echocardiography showed a hypoechoic mass in the right atrium (RA), pericardial thickening, and a left ventricular ejection fraction of 44% with reduced ventricular wall motion **(A)**. Contrast-enhanced ultrasound showed a filling defect in the RA and no wash-in of the hypoechoic mass **(B)**. Arterial phase computed tomography angiography showed that the mass (measuring 3.7 × 3.0 cm) was located on the right posterior wall of the RA and had a broad base **(C)**. In Case 2, transthoracic echocardiography showed a mass with medium-to-low echogenicity, with a broad basal change and an unclear border with the posterior wall of the RA, that extended toward the opening of the superior vena cava. The LVEF was measured at 60% with normal wall movement **(D)**. Contrast-enhanced ultrasound showed a filling defect in the RA with a moderately enhancing mass **(E)**. Computed tomography angiography showed a hypodense right atrial mass (measuring 7.9 × 6.0 cm) with heterogeneous enhancement and pericardial thickening **(F)**.

**Figure 2:**
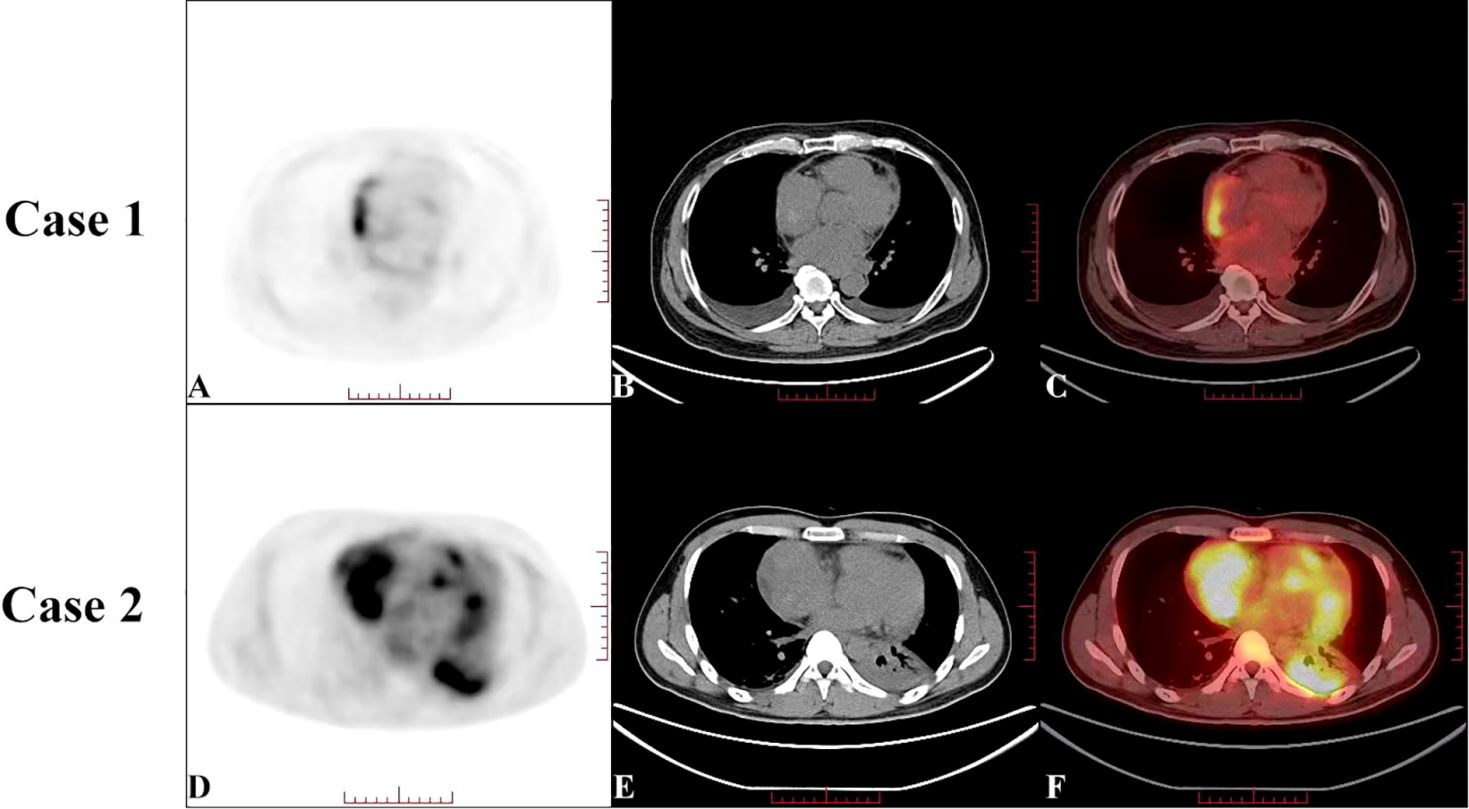
Preoperative positron emission tomography-computed tomography (PET-CT) of Case 1 **(A–C)** showed an increase in radiotracer uptake in an arcuate-shaped area in the outer wall of the right atrium, with a maximum standard uptake value (*SUV*_max_) of 7.29. The hypodense mass showed no significant increase in radiotracer uptake on the PET scan. Preoperative PET-CT of Case 2 **(D–F)** showed a significant increase in radiotracer uptake in the right atrial mass, with a *SUV*_max_ of 9.11, which was highly suggestive of cardiac angiosarcoma.

A 59-year-old Chinese American male with no prior comorbidities, 179 cm in height and weighing 79 kg, presented to the emergency department with a 4-month history of progressive chest tightness and bilateral lower extremity edema. Upon admission, the patient exhibited acute respiratory distress accompanied by chest pain and lower extremity edema. Electrocardiography (ECG) showed paroxysmal atrial fibrillation (AF), while TTE showed a hypoechoic mass in the right atrium (RA), pericardial thickening, and a left ventricular ejection fraction (LVEF) of 44% with reduced ventricular wall motion (**Figure 1A**, **Online Supplementary Video 1**). CEUS further confirmed a filling defect in the RA and an absence of wash-in within the hypoechoic mass (**Figure 1B**, **Online Supplementary Video 2**). Both TEE and CTA showed that the mass (measuring 3.0 × 3.7 cm) was located on the right posterior wall of the RA, had a broad base, and involved the superior and inferior vena cava (**Figure 1C**, **Online Supplementary Video 3**). CTA also showed hypodense filling defects without enhancement as well as pericardial thickening. A whole-body PET scan revealed an arcuate-shaped increase in radiotracer uptake in the lateral wall of the RA with a maximum standard uptake value (*SUV*_max_) of 7.29, while the hypodense mass displayed no significant increase in radiotracer uptake in the PET scan (**Figures 2A–C**). The patient’s creatine kinase (CK) and CK-MB levels were 183 and 11.4 U/L, respectively. The level of lactate dehydrogenase (LDH) was 213 U/L, that of troponin T (TNT) was 0.015 µg/L, and that of N-terminal pro-B-type natriuretic peptide (NT-proBNP) was 915.8 ng/L.

**Figure 3:**
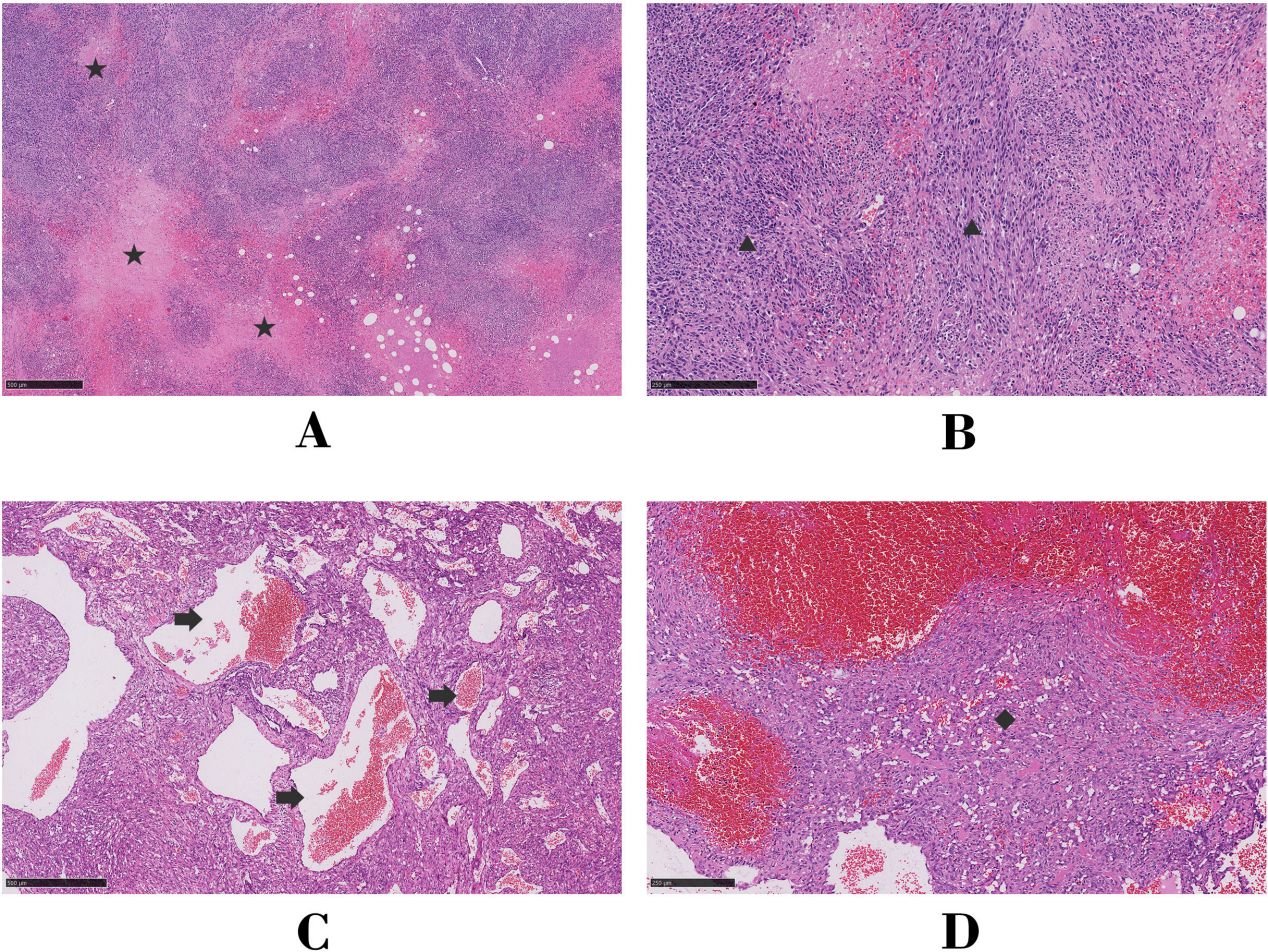
Microscopic analysis of hematoxylin and eosin-stained histologic sections for Case 1 **(A, B)** and Case 2 **(C, D)**. Asterisk, focal necrosis; triangle, fusiform tumor cells; arrow, tortuous vessels of the tumor; diamond, solid epithelioid tumor cells.

Given the increase in radioactivity in an arcuate-shaped area in the lateral wall of the RA with no significant increase in mass, elevated D-dimer or fibrinogen levels, and low serologic tumor marker levels, the atrial mass was suspected to be a thrombus. The arcuate-shaped increase in radiotracer uptake was attributed to right atrial wall inflammation, possibly related to AF (1, 2). After 2 months of thrombolytic therapy, the patient was readmitted to the hospital for chest tightness, chest pain, recurrent lower extremity edema, and heart failure. A repeat TTE showed a LVEF of 62%, diastolic dysfunction, pericardial thickening, and a hypoechoic mass without significant size reduction. Based on these findings, constrictive pericarditis and right atrial thrombosis were considered, and surgery was performed with a cardiopulmonary bypass time of 325 minutes. During surgery, significant thickening of the posterior wall of the RA (maximum thickness of approximately 40 mm) was observed, along with diffuse thickening of the pericardium. The mass was partially resected. Pathologic examination of the atrial tissue cross-section revealed hemorrhage and necrosis, with diffuse tumor growth and typical vascular areas. The tumor consisted of a combination of solid, epithelioid cells and spindle-shaped cells, with frequent mitotic figures (**Figures 3A, B**). Immunohistochemical analysis showed positivity for CD34, CD31, ERG, FLi-1, Ki-67, vimentin, EMA, and INI-1 and negativity for CK, CK7, SMA, S-100, and desmin. Based on these findings and the patient’s medical history, a diagnosis of CA was made. The patient died 8 days after surgery due to hemodynamic instability and the inability to maintain blood pressure.

### 2.2 Case 2

A 27-year-old man, 170 cm in height and weighing 67.5 kg, presented to his local hospital with dyspnea and cough. Both TTE and enhanced CT showed significant pericardial effusion and a mass in the RA. Despite undergoing pericardial puncture drainage, his symptoms persisted, and he was transferred to our hospital for further management.

On arrival, his TTE showed a mass in the RA with mid-to-low echogenicity, a broad base, and an unclear boundary with the posterior RA wall. This mass extended toward the superior vena cava orifice, resulting in increased blood flow velocity in the superior vena cava. The LVEF was measured at 60% and wall movement was normal (**Figure 1D**, **Online Supplementary Video 4**). CEUS revealed a filling defect in the RA with a moderately enhancing mass (**Figure 1E**, **Online Supplementary Video 5**). CT showed a hypodense mass in the RA (7.9 × 6.0 cm) with heterogeneous enhancement and pericardial thickening (**Figure 1F**, **Online Supplementary Video 6**). PET revealed a significant increase in radiotracer uptake within the right atrial mass with a *SUV*_max_ of 9.11, highly suggestive of CA (**Figures 2D–F**). The patient’s CK-MB level was 12.0 U/L. The concentrations of CK, LDH, TNT, and NT-proBNP were 169 U/L, 205 U/L, 0.02 μg/L, and 875.9 ng/L, respectively.

The patient underwent radical resection surgery, during which extensive adhesions between the pericardium and the RA were identified. The cardiopulmonary bypass time of the surgery was 115 minutes, with a cross-clamp time of 73 minutes. The tumor extended superiorly to parts of the superior vena cava, inferiorly to the inferior vena cava orifice, posteriorly to the anterior edge of the atrial sulcus, and left to the anterior edge of the tricuspid annulus. Pathologic examination of the atrial tissue cross-section showed no hemorrhage or necrosis; however, irregular vascular lacunar-like structures could be observed, along with characteristic blood cavities and lakes covered by spindle-shaped endothelial cells. In some areas, endothelial cells were highly proliferative, showing clustered or papillary growth with minimal mitotic figures (**Figures 3C, D**). Immunohistochemical analysis showed positivity for CD34, CD31, ERG, Ki-67, SMA, and INI-1 and negativity for CK, HMB45, S-100, desmin, and Mrlan-A. Two weeks after surgery, TTE confirmed the disappearance of the original right atrial mass, unobstructed flow in both the superior and inferior vena cava, and a normal RA volume. Enhanced CT images showed no significant metastasis. Monthly follow-up measurements remained stable for eight months after discharge.

## 3 Discussion

Angiosarcoma is a rare and aggressive soft tissue tumor originating from endothelial cells of blood or lymphatic vessels. It is commonly detected in the extremities, particularly the lower limbs, followed by the trunk, head, and neck. CA is exceptionally rare, accounting for 25%–30% of all primary malignant cardiac tumors, with the RA being the most frequent site of origin (3). The incidence of CA is higher in males (63.1%) than females, with a median age at onset of 50.5 years (4). CA often presents with nonspecific clinical symptoms such as dyspnea, chest pain, fever, and pericardial effusion, which may result from its aggressive nature. Up to 80% of cases initially present with pericardial infiltration, potentially leading to misdiagnosis as constrictive pericarditis (5). Pericardial effusion drainage and cytological biopsy have limited diagnostic success rates for CA (6) and the gold standard for diagnosis remains immunohistochemical analysis of tissue specimens, with markers of positivity including CD31, CD34, ERG, FLI-1, and von Willebrand factor. Among these factors, FLI-1 and ERG demonstrate superior specificity and sensitivity (7, 8).

On two-dimensional echocardiography, CA typically appears as a nodular or lobulated hypoechoic mass. RA-originating CA often presents with a broad-based attachment, accompanied by pericardial effusion or thickening (9). In Case 1, CA invasion into the pericardium caused diffuse thickening and adhesion, leading to restricted movement of the ventricular free wall. In Case 2, the RA mass invaded the superior vena cava, resulting in increased blood flow velocity at the vena cava entrance. Although the diagnostic value of echocardiography for CA is limited, its specificity for detecting intracardiac tumors is as high as 95%, making it the preferred screening method for cardiac tumors (10).

Contrast-enhanced ultrasound provides critical insights into the perfusion characteristics of tumors and serves as a valuable tool for differentiating benign from malignant lesions (11). In Case 1, the complete perfusion defect initially led to misdiagnosis as an atrial thrombus. Notably, 3.6% of malignant tumors exhibit reduced perfusion due to ischemic necrosis (12), and, in Case 1, the perfusion defect was attributed to extensive intratumoral hemorrhaging and necrosis, a hallmark of poorly differentiated CA (8). In contrast, Case 2 demonstrated moderate heterogeneous enhancement on CEUS, consistent with the histopathological findings of abundant irregular vascular lacunae and minimal necrosis. The striking disparity in CEUS enhancement patterns between the two cases (complete perfusion defect *vs*. moderate heterogeneous enhancement) underscores the significant impact of tumor vascular architecture on perfusion characteristics.

On contrast-enhanced CT, CA typically presents as a homogeneous or heterogeneously dense mass with broad-based attachment and infiltrative margins (13). Characteristic filling defects are observed when the tumor involves the tricuspid annulus or cavoatrial junctions (14). Here, Case 1 exhibited a non-enhancing hypodense lesion initially mistaken for a thrombus, while Case 2 demonstrated heterogeneous enhancement, aligning with classical CA features. These contrasting enhancement patterns—non-enhancing *versus* heterogeneously hyperenhancing—reflect the stark differences in tumor necrosis and vascular supply between these cases. Furthermore, as previously mentioned, the enhancement patterns on CEUS and CT were consistent in both cases, highlighting their complementary roles in characterizing CA vascularity.

Fluorodeoxyglucose (^18^F-FDG) PET/CT is a crucial imaging modality for assessing tumor metabolic activity (15). Yamakuni et al. established a diagnostic threshold of *SUV*_max_ ≥3.5, demonstrating 100% sensitivity and 80% specificity for CA diagnosis (16). In Case 1, focal arcuate-shaped uptake (*SUV*_max_ 7.29) was observed in the RA lateral wall, suggestive of localized hypermetabolic activity. Wan et al. detected potential associations between atrial wall hypermetabolism and atrial fibrillation (1, 2). Given the presence of atrial fibrillation, elevated D-dimer levels, and the absence of significant metabolic activity in the RA mass, the initial diagnosis favored thrombus. However, postoperative pathological analysis revealed that the tumor primarily consisted of hemorrhagic and necrotic tissue, with atrial wall thickening and increased metabolism attributable to tumor infiltration. In contrast, Case 2 demonstrated intense heterogeneous uptake (*SUV*_max_ 11) within the pericardium-invading mass, consistent with typical CA findings. The marked metabolic heterogeneity between the two cases paralleled the disparities observed on CEUS and contrast-enhanced CT. Notably, Case 1 exhibited low Ki-67 levels, which correlate with the cell division cycle and differentiation status, indicative of poor differentiation (8, 17) and potentially explaining the hypometabolic phenotype.

Owing to the marked heterogeneity in CA differentiation (18), multimodal imaging analysis combining contrast-enhanced and PET metabolic patterns reveals diverse phenotypic combinations, including hyperenhancement with hypermetabolism, hypoenhancement with hypermetabolism, heterogeneous enhancement with hypermetabolism, and heterogeneous enhancement with heterogeneous metabolism. We present the first report of two CA cases exhibiting contrasting enhancement-metabolism patterns, including a rare hypoenhanced-hypometabolic phenotype (Case 1) that has not been previously documented in the literature. This striking dichotomy not only expands the imaging spectrum of CA but also highlights the considerable diversity of enhancement-metabolism pattern combinations.

## 4 Conclusion

CA is a rare yet highly aggressive malignancy that poses significant diagnostic challenges owing to its atypical clinical presentation. The two cases reported here demonstrated strikingly contrasting enhancement-metabolism patterns (hypoenhanced-hypometabolic *vs*. hyperenhanced-hypermetabolic), reflecting not only the diversity of imaging phenotypes and metabolic patterns but also providing critical insights into the underlying biological heterogeneity of CA.

## Data Availability

The original contributions presented in the study are included in the article/**Supplementary Material**. Further inquiries can be directed to the corresponding authors.
